# Effects of *Yerba maté*, a Plant Extract Formulation (“YGD”) and Resveratrol in 3T3-L1 Adipogenesis

**DOI:** 10.3390/molecules191016909

**Published:** 2014-10-21

**Authors:** Juliana C. Santos, Érica M. F. Gotardo, Mitsue T. Brianti, Mahmood Piraee, Alessandra Gambero, Marcelo L. Ribeiro

**Affiliations:** 1 Laboratory of Microbiology and Molecular Biology, Clinical Pharmacology and Gastroenterology Unit, Sao Francisco University Medical School, Bragança Paulista-SP 12916-900, Brazil; E-Mails: ju_carvalho_s@hotmail.com (J.C.S.); erikinha_gotardo@yahoo.com.br (E.M.F.G.); mitsue.brianti@gmail.com (M.T.B.); alessandra.gambero@usf.edu.br (A.G.); 2 Groupe Persavita Inc., CP 49586 DU-MUSEE, Montreal, QC H3T 2A5, Canada; E-Mail: mpiraee@persavita.com

**Keywords:** *Yerba maté*, resveratrol, adipogenesis

## Abstract

We aimed to evaluate the *in vitro* effects of *yerba maté*, YGD (a herbal preparation containing *yerba maté*, guarana and damiana), and resveratrol on adipogenesis. The anti-adipogenic effects of *yerba mate*, YGD, resveratrol and YGD + resveratrol and *yerba mate* + resveratrol combinations were evaluated in 3T3-L1 cells by Oil Red staining, cellular triglyceride content, and PCR quantitative array. The results demonstrated that all of the tested compounds inhibited adipogenesis. *Yerba maté* extract significantly down-regulated the expression of genes that play an important role in regulating adipogenesis, such as *Adig*, *Axin*, *Cebpa*, *Fgf10*, *Lep*, *Lpl*, and *Pparγ2*. In addition, these genes, YGD also repressed *Bmp2*, *Ccnd1*, *Fasn*, and *Srebf1*. Resveratrol also modulated the expression of *Adig*, *Bmp2*, *Ccnd1*, *C/EBPα*, *Fasn*, *Fgf10*, *Lep*, *Lpl*, and *Pparγ2*. Moreover, resveratrol repressed *Cebpb*, *Cdk4*, *Fgf2*, and *Klf15*. The *yerba maté* extract and YGD up-regulated the expression of genes involved in inhibiting adipogenesis, such as *Dlk-1*, *Klf2*, and *Ucp1*. Resveratrol also induced the expression of *Klf2* and *Ucp1*. In addition resveratrol modulated the *Ddit3*, *Foxo1*, *Sirt1*, and *Sirt2*. The combined effects of these compounds on gene expression showed similar results observed from individual treatments. Our data indicates that the synergy between the compounds favors the inhibition of adipogenesis.

## 1. Introduction

The high prevalence of obesity created a major public health concern because of the associated weight-related diseases that result in significant morbidity and mortality and reduced quality of life [1]. The increasing incidence of obesity suggests that this epidemic will continue to grow [2]. The global anti-obesity strategies focus on dietary and lifestyle modifications, *i.e.*, restricting caloric intake and increasing physical activity, to slow down the development of obesity [3]. Research in the nutrition field has recently aroused considerable interest based on the potential of natural products to counteract obesity and the associated health complications [4,5].

Several studies have identified *yerba maté (Ilex paraguariensis)* as an excellent candidate in the struggle against obesity [6,7,8,9,10,11,12,13,14,15,16,17]. Data obtained from experiments conducted in diet-induced obesity models have shown that *yerba maté* suppresses body weight gain and visceral fat accumulation and decreases serum levels of cholesterol, triglycerides, LDL cholesterol, glucose, insulin, pancreatic lipase and leptin [6,10,14,15,16,17,18,19,20]. The molecular mechanisms by which yerba maté regulates obesity have also been studied. *In vitro* and *in vivo* studies have shown that *yerba maté* modulates signaling pathways, which regulate adipogenesis, antioxidant, anti-inflammatory and insulin signaling responses [6,7,8,9,10,11,12,14,15,16,17,21,22].

The anti-obesity role of *yerba maté* was also evaluated in humans. In a clinical study, it has been demonstrated that a herbal preparation containing *yerba maté*, Guarana and Damiana (“YGD”) significantly delayed gastric emptying, reduced the time to perceived gastric fullness, and induced significant weight loss over 45 days in overweight patients [23]. Subsequently, YGD has been demonstrated to produce a robust acute effect on caloric intake and eating duration, suggesting YGD strengthens within-meal satiation, an effect that may be mediated by changes in gastric emptying time [24].

Another important candidate to manage obesity is resveratrol (3,4,5-trihydroxystilbene), a naturally occurring polyphenol found in different plant species [25]. Resveratrol was first studied because of its cardioprotective effects. Throughout the years, epidemiological studies have shown that due to its high content of resveratrol, a moderate intake of wine, especially red wine, reduces the risk of cardiovascular disease [26]. Besides on initial cardioprotective effects, further on the anti-cancer, anti-inflammatory, antioxidant, and anti-obesity properties of resveratrol have been also studied and characterized [27,28,29,30].

Several *in vitro* and animal studies have demonstrated that resveratrol has an antiobesity potential by inhibiting adipocyte differentiation, decreasing proliferation, inducing apoptosis, decreasing lipogenesis, and promoting lipolysis and FA β-oxidation [30,31,32,33,34,35,36,37,38,39,40,41,42]. Additionally, clinical studies have shown antiobesity potential for resveratrol in obese volunteers [43,44,45,46]. Taken together, the data from clinical studies, *in vitro* and animal data indicate that the potential antiobesity effects and health promoting effects of resveratrol in the context of obesity should be better comprehended.

Considering the importance of adipogenesis in the obesity, the present study aimed to evaluate *the in vitro* effects of *yerba maté*, YGD, and resveratrol on expression level of key genes involved in the process of adipogenesis.

## 2. Results and Discussion

The MTT analysis revealed that YGD *yerba maté* and resveratrol were not cytotoxic to 3T3-L1 cells. Concerning the YGD + resveratrol and *yerba maté* + resveratrol mixtures the MTT revealed they were not cytotoxic.

**Figure 1:**
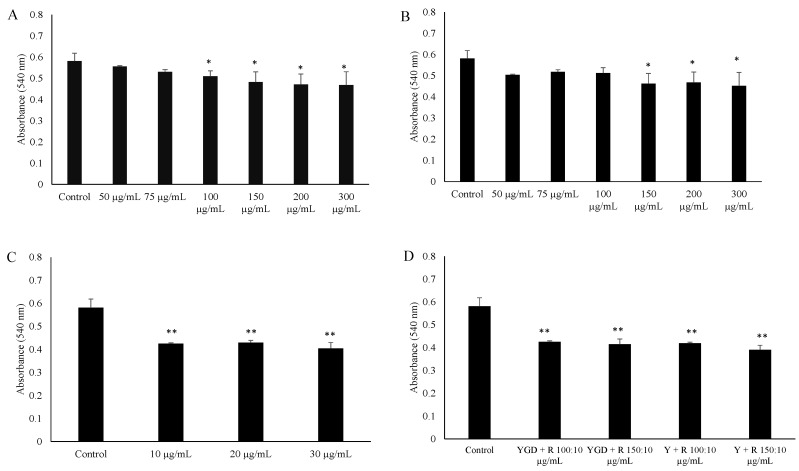
Effect of (*yerba maté*, guarana and damiana) YGD (**A**); *yerba maté* (**B**); resveratrol (**C**) or mixed compounds (**D**) on adipogenesis in 3T3-L1 cells according to Oil Red O Assay. * *p* < 0.05 and ** *p* < 0.01 compared with control group.

**Figure 2:**
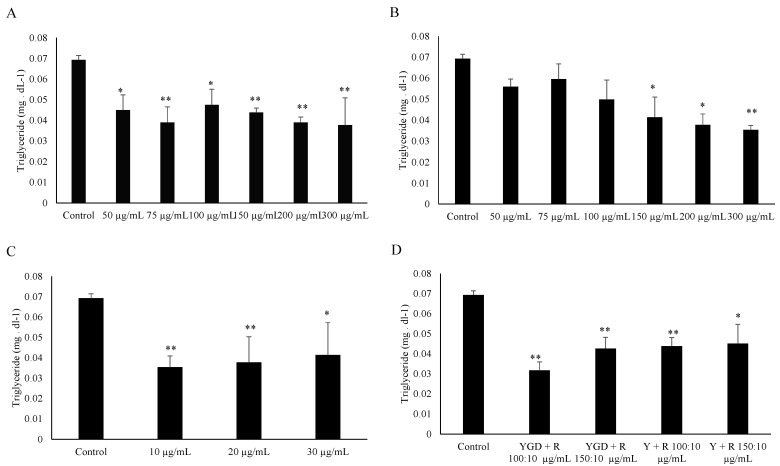
Effect of YGD (**A**); *yerba maté* (**B**); resveratrol (**C**) or mixed compounds (**D**) on adipogenesis in 3T3-L1 cells according to evaluation of triglyceride content. * *p* < 0.05 and ** *p* < 0.01 compared with control group.

**Figure 3:**
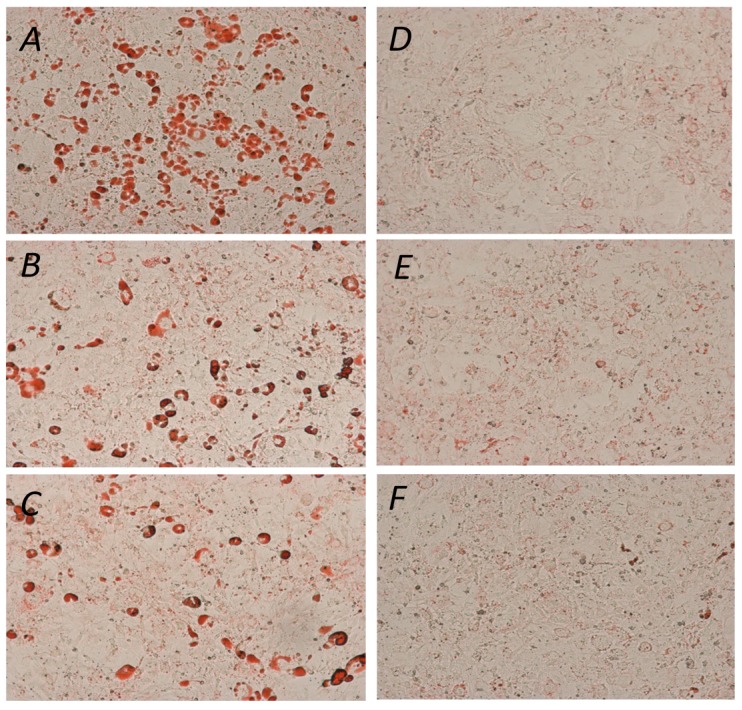
Effect of *yerba maté*, YGD and resveratrol on 3T3-L1 differentiation. (**A**) Control; (**B**) YGD (100 μg/mL); (**C**) *yerba maté* (150 μg/mL); (**D**) resveratrol (10 μg/mL); (**E**) YGD + resveratrol (100:10 μg/mL); and (**F**) *yerba maté* + resveratrol (150:10 μg/mL). Adipogenic differentiation was assessed using Oil Red O staining for lipid droplets 100 ×.

The ability of YGD, *yerba maté* (50, 75, 100, 150, 200 or 300 μg/mL) or resveratrol (10, 20 or 30 μg/mL) to prevent lipid accumulation was examined by Oil Red O staining and evaluation of triglyceride content of 3T3-L1 adipocytes. Our data indicated that all of the tested compounds inhibited adipogenesis, but at different concentrations. YGD inhibited adipogenesis in both methods at a concentration of 100 µg/mL (Figure 1A and Figure 2A). *Yerba maté* significantly inhibited adipogenesis at a concentration of 150 µg/mL (Figure 1B and Figure 2B). Resveratrol had inhibitory activity at the lowest concentration tested (Figure 1C and Figure 2C). Next the effects of YGD + resveratrol was evaluated (100:10 or 150:10 μg/mL), or *yerba maté* + resveratrol (150:10 or 200:10 μg/mL) in the inhibition of adipogenesis. Our data also showed that all of the tested combination inhibit the adipogenesis (Figure 1D and Figure 2D). The Figure 3 illustrates the significative results obtained from Oil Red O staining.

The anti-adipogenic effects occurred at concentrations that did not affect cell viability. Regarding *yerba maté*, our data indicated that it inhibited adipogenesis, as previously described before [12,14]. The YGD also regulated this process negatively, but at a lower concentration. We could infer that this result might be explained by the presence of guarana and damiana in the YGD formulation. However, there is no evidence in the literature showing the anti-adipogenic effects of these compounds. Resveratrol also showed an effect on adipogenesis as reported previously [31,47]. The combined effects of these compounds on adipogenesis were also evaluated. The data presented shows that both combinations (YGD + resveratrol, and *yerba maté* + resveratrol) inhibited adipogenesis at the lowest concentration. It is noted that the level of inhibition of adipogenesis with the combinations is higher than that observed for resveratrol alone. The results are attributable to a synergistic effect obtained from the potent anti-adipogenic effect of resveratrol in combination with the other compounds.

To determine the effect of YGD (100 μg/mL), *yerba maté* (150 μg/mL), resveratrol (10 μg/mL), YGD + resveratrol (100:10 μg/mL), or *yerba maté* + resveratrol (150:10 μg/mL) on the expression of several genes that regulate adipogenesis, the Mouse Adipogenesis RT^2^ Profiler^TM^ PCR Array (SABiosciences) was utilized. This assay was performed at the lowest concentrations in which an inhibition of adipogenesis by both employed methods was observed. The triplicate samples from each group gave reproducible results.

The results from the PCR array revealed that the *yerba maté* extract modulated the expression of 12% (10/84) the genes. YGD modulated the expression of 17% (14/84) of the genes. Although YGD modulates the expression of more genes than *yerba maté*, we noted that both compounds act in the same pathways of adipogenesis (Table 1). Concerning the resveratrol effects on adipogenesis, our data showed that this compound modulated the expression of 24% (20/84) of the genes. Among these genes, 10 were exclusively modulated by resveratrol that were not modulated by other test compounds. Thus, these data allow us to infer that resveratrol could act in adipogenesis in a more comprehensive way and somewhat distinct from the *yerba maté* based compounds. The analysis of tested mixtures is also shown in Table 1. Our data indicated that the synergic effects of the combination resveratrol -*yerba maté* or -YGD might be responsible for the modulation of all studied genes.

The *Yerba maté* extract significantly down-regulated the expression of genes that play an important role in regulating adipogenesis, including *Adig* (Adipogenin), *Axin* (Axin 1), *Cebpa* (CCAAT/enhancer binding protein (C/EBP) alpha), *Fgf10* (Fibroblast growth factor 10), *Lep* (Leptin), *Lpl* (Lipoprotein lipase), and *Pparγ2* (Peroxisome proliferator-activated receptor, gamma 2). In addition to those genes, YGD also down-regulated *Bmp2* (Bone morphogenetic protein 2), *Ccnd1* (Cyclin D1), *Fasn* (Fatty acid synthase), and *Srebf1* (Sterol regulatory element binding transcription factor 1). As observed for *yerba maté* and/or YGD, resveratrol also modulated the expression of *Adig*, *Bmp2*, *Ccnd1*, *C/EBPα*, *Fasn*, *Fgf10*, *Lep*, *Lpl*, and *Pparγ2*. Moreover, resveratrol repressed *Cebpb* (CCAAT/enhancer binding protein (C/EBP), beta), *Cdk4* (Cyclin-dependent kinase 4), *Fgf2* (Fibroblast growth factor 2), and *Klf15* (Kruppel-like factor 15). The *yerba maté* extract and YGD up-regulated the expression of genes involved in inhibiting adipogenesis, such as *Dlk-1* (Delta-like 1 homolog), *Klf2* (Kruppel-like factor 2), and *Ucp1* (Uncoupling protein 1). Resveratrol also induced the expression of *Klf2* and *Ucp1*. Additionally, resveratrol modulated the *Ddit3* (DNA-damage inducible transcript 3), *Foxo1* (Forkhead box O1), *Sirt1* (Sirtuin 1), and *Sirt2* (Sirtuin 2) gene expression levels. The combined effects of these compounds on gene expression showed similar results on expression level of targeted genes.

**Table 1:** Fold regulation results from the PCR array.

Functional Gene Group	Gene	*Yerba maté* 150 μg/mL	YGD 100 μg/mL	Resveratrol 10 μg/mL	YGD 100 + R 10 μg/mL	Y 150 + R 10 μg/mL
Pro-adipogenic	*Adig*	−2.08 ± 0.31 *	−2.46 ± 0.93 *	−2.81 ± 0.32 *	−2.04 ± 0.47 *	−2.43 ± 0.59 *
	*Axin*	−2.20 ± 0.83 *	−2.80 ± 0.86 *	−1.58 ± 0.11	−2.99 ± 0.68 *	−2.60 ± 0.51 *
	*Bmp2*	−1.95 ± 0.54	−4.20 ± 1.58 *	−2.00 ± 0.11 *	−3.65 ± 0.94 *	−2.01 ± 0.93 *
	*Cebpa*	−4.13 ± 0.63 *	−7.20 ± 2.11 *	−2.29 ± 0.31 *	−5.58 ± 1.58 *	−4.41 ± 1.48 *
	*Cebpb*	−1.04 ± 0.14	−1.24 ± 0.+23	−2.28 ±0.88 *	−2.69 ± 0.69 *	−2.61 ± 0.62 *
	*Ccnd1*	−1.92 ± 0.58	−2.17 ± 0.83 *	−2.65 ± 0.65 *	−-2.00 ± 0.65 *	−2.06 ± 0.65 *
	*Cdk4*	1.12 ± 0.44	1.81 ± 0.28	−2.39 ± 0.65 *	−2.88 ± 0.69 *	−3.74 ± 0.77 *
	*Fasn*	−1.97 ± 0.18	−2.53 ± 0.64 *	−2.31 ±0.48 *	−2.52 ± 0.82 *	−2.22 ± 0.12 *
	*Fgf2*	−1.41 ± 0.09	−1.34 ± 0.49	−2.36 ±0.81 *	−2.45 ± 0.51 *	−3.25 ± 0.31 *
	*Fgf10*	−3.19 ± 0.69 *	−3.59 ± 1.64 *	−4.89 ± 1.94 *	−2.74 ± 0.34 *	−3.55 ± 0.84 *
	*Klf15*	−1.25 ± 0.21	−1.88 ± 0.22	−3.83 ± 0.97 *	−4.55 ± 0.94 *	−4.77 ± 0.84 *
	*Lep*	−3.32 ± 0.45 *	−4.52 ± 1.21 *	−2.07 ± 0.41 *	−2.21 ± 0.61 *	−2.38 ± 0.96 *
	*Lpl*	−2.94 ± 0.69 *	−6.79 ± 1.65 *	−7.03 ± 0.91 *	−6.21 ± 2.01 *	−4.09 ± 1.19 *
	*Pparg*	−2.02 ± 0.22 *	−3.08 ±1.05 *	−2.41 ± 0.89 *	−2.69 ± 0.88 *	−2.24 ± 0.51 *
	*Srebf1*	−1.87 ± 0.35	−2.97 ± 1.18 *	-1.06 ± 0.58	−2.32 ± 0.76 *	−2.88 ± 0.83*
Anti-adipogenic	*Ddit3*	1.00 ± 0.14	1.47 ± 0.72	2.47 ± 0.57 *	2.51 ± 0.40 *	2.73 ± 0.52 *
	*Dlk1*	4.82 ± 1.42 *	1.01 ± 0.23	1.44 ± 0.81	2.98 ± 0.88 *	3.61 ± 0.89 *
	*Foxo1*	1.87 ± 0.50	1.78 ± 0.29	2.30 ± 0.88 *	3.54 ± 0.92 *	3.65 ± 0.91 *
	*Klf2*	3.91 ±1.06 *	4.82 ± 1.14 *	5.57 ± 1.28 *	3.65 ± 0.55 *	4.63 ± 0.73 *
	*Ucp1*	3.47 ± 1.59 *	4.15 ± 1.67 *	6.29 ± 2.16 *	6.02 ± 1.99 *	6.80 ± 2.73 *
	*Sirt1*	1.23 ± 0.13	1.30 ± 0.50	2.94 ± 0.88 *	3.20 ± 1.02 *	3.08 ± 0.99 *
	*Sirt2*	1.79 ± 0.48	1.52 ± 0.22	2.08 ± 0.27 *	2.88 ± 0.92 *	2.76 ± 0.89 *

* Significative down-regulation was considered when the fold regulation was ≤−2; significative up-regulation was considered when the fold regulation was ≥+2.

Adipogenesis is the developmental process by which a multipotent mesenchymal stem cell differentiates into a mature adipocyte. This process involves a highly regulated and coordinated cascade of transcription factors, including members of the PPAR, C/EBP, and SREBP families, which together lead to the establishment of the differentiated state [48]. In this context, it has been observed that *yerba maté* and resveratrol modulates adipogenesis by down regulating the levels of pro-adipogenic transcription factors, such as *Pparγ2* and *Cebpa*, *in vivo* [6,14,23,33,35,37,38]. Similarly, our data also shows that *yerba maté*, YGD, resveratrol, and their combinations were able to down-regulate the expression of *Pparγ2* and *Cebpa*. Additionally, *Cebpb* was repressed by resveratrol, as previously described [36], and by the mixed compounds.

SREBP1 is a transcription factor that controls fatty acid synthase, and functions an additional regulator of adipogenesis in parallel with C/EBPα and PPARγ pathways [49]. Here, we demonstrated that YGD and the mixed compounds down-regulated the downstream target gene *Fasn. Fasn* was also down regulated by resveratrol as previously described [50]. The data presented in this study also show that the tested compounds were able to repress *Adig* and *Srebf1*, adipogenic markers that mediate the initiation of adipogenesis.

Numerous effects of the KLF protein family have been reported in different tissues. In the 3T3-L1 cell line model, KLF2 inhibits adipogenesis by inhibiting PPARγ [51]. By assessing the expression of the *Klf2*, it was shown that all tested compounds increased the expression level of this gene, which may then contribute to the observed repression of *Pparγ_2_* expression. Similar results were previously observed for *yerba maté* [14]. On the other hand, KLF15 plays an essential role in adipogenesis promoting the differentiation of 3T3-L1. It has been proposed that the cooperative effects of KLF15, PPARγ, and C/EBPα are responsible for the full range of adipocyte-specific gene expression during the differentiation process of adipocytes [52]. Our gene expression data showed that resveratrol and the mixed compounds down-regulated the expression of *Klf15*, further contributing to the inhibition of adipogenesis.

In addition, expression of C/EBPs and PPARγ is dependent on the activation of other genes that are essential for adipogenesis such as *Dlk1*.It has been reported that this gene contributes to inhibition of adipogenesis via inhibitory actions on C/EBP-β and C/EBP-δ in the pre-adipocyte cell line 3T3-L1. It is believed that down-regulation of *Dlk1* is critical for adipogenesis to occur [53,54]. We observed that *yerba maté*, as previously described [14], and the combination of compounds tested in this study up-regulated *Dlk1*, which might be at least in part, responsible for the observed inhibition of adipogenesis. The results presented in this study show that all of the tested compounds down regulated the *Pparγ* and *Cebpa* mRNA expression, which leads to further down regulation of other genes such as *Lep* and *Lpl*. Similar data were previously observed for *yerba maté* [7] and resveratrol [30].

Bone morphogenetic proteins (Bmps), are members of the transforming growth factor-β (Tgf-β) family, which regulate diverse physiological processes including adipose tissue formation [55]. It has been demonstrated that Bmp activity prior to differentiation induction is responsible for adipocyte differentiation. Thus, it has been proposed that BMP-2 may act as a potent adipogenic agent if presented together with activators of PPARγ [56]. The data presented in this work shows that YGD, resveratrol and the mixed compounds were able to significantly down-regulate this pro-adipogenic gene.

FOXO1 is a transcription factor which negatively regulate the adipogenesis [57,58] . It has been shown that FOXO1 can bind to PPARγ promoter region and suppress its expression [58]. Complementarily, SIRT1 and SIRT2 have been shown to regulate the adipogenesis through modulation of FOXO1 acetylation/phosphorylation activity and may play a role in the inhibition of adipocyte differentiation [59,60]. It has been observed that resveratrol is an indirect activator of SIRT1 and SIRT2 leading to the suppression of adipogenesis [61,62]. Thus, the up-regulation of *Sirt1*, *Sirt2* and *Foxo1* by resveratrol and its combination with yerba mate or YGD observed in this study might contribute to the exhibited down-regulation *of Pparg2*.

It has been described that *Ddit3* (also known as *Chop-10*) is a dominant-negative member of the C/EBP family. It is initially expressed by growth-arrested preadipocytes and sequesters/inactivates C/EBPβ during 3T3-L1 preadipocyte differentiation. Thus, its down-regulation is required for the complete adipocyte differentiation [63,64]. Therefore, the up-regulation observed after resveratrol and mixed compounds treatment might be one of the mechanisms by which these compounds exert their antiadipogenic effect. Another C/EBPβ regulator is *Fgf10*, which plays crucial roles in adipogenesis by contributing to the expression of C/EBPβ through an autocrine/paracrine mechanism [65]. Although our data shows that all of the tested products were able to significantly down-regulate *Fgf10*, a direct effect was observed only for resveratrol and mixed compounds. Additionally, *Cdk4* activity has been proposed to be required for adipogenesis. *Cdk4* participates in the clonal expansion phase of adipocyte differentiation through activation of PPARγ [66]. Our data showed that resveratrol and the mixed compounds were able to down-regulate *Cdk4*, which might contribute to the *Pparg* repression observed.

It has been proposed that WNT/β-catenin pathway exert a negative regulation on adipogenesis [67]. When the WNT/β-catenin pathway is inactive, β-catenin is proteasomally degraded by the destruction complex composed of adenomatous polyposis coli (APC), glycogen synthase kinase (GSK)3β and AXIN [68]. Activation of the WNT/β-catenin pathway induces the expression of its target genes including cyclin D1 (CCND1), which has been reported to down-regulate PPARγ, a major adipogenic transcription factor [69]. In our study, *Axin,* which is known to be a negative regulator of the WNT/β-catenin pathway, was down-regulated by *yerba maté*, YGD and the combination of compounds. Regarding *Ccnd1*, our data show an inhibition of its expression after YGD, resveratrol and mixed compound treatments. These results are in accordance with a previous study, which demonstrated an early effect of resveratrol on *Ccnd1* down-regulation. The authors also observed a subsequent inhibition of cell cycle reentry and clonal expansion [70], which leads to an inhibition of adipogenesis.

*Ucp1* is known to be a significant component of whole body energy expenditure, and its dysfunction contributes to the development of obesity. In this study, we observed that all of the tested compounds were able to up-regulate the expression of *Ucp1*. Similar results were previously described for *yerba maté* [6,7] and resveratrol [39,71].

## 3 Experimental Section

### 3.1. Materials

The soluble *yerba maté* powder and the patented herb extract formulation YGD (Zotrim, which contains 46% *yerba mate*, 39% guarana and 15% damiana extracts) were provided by Natures Remedies, Amersham, UK. Resveratrol and Oil Red O were purchased from Sigma-Aldrich (St. Louis, MO, USA). Cell culture reagents were purchased from Invitrogen (Invitrogen Life Technologies, Alameda, CA, USA).

### 3.2. In Vitro Evaluation of Adipogenesis

#### 3.2.1. 3T3-L1 Cell Culture

The 3T3-L1 cell line was purchased from the Rio de Janeiro Cell Bank (Rio de Janeiro, Brazil) and it was cultured to confluence in DMEM supplemented with 10% fetal bovine serum and 10 mL/L penicillin/streptomycin at 37 °C and 5% CO_2_. Forty-eight hours after achieving confluence (day 0), the cells were incubated in differentiation medium (DMEM supplemented with 10% fetal bovine serum, 0.25 μM dexamethasone, 10 μg/mL insulin and 0.5 mM IBMX). After 96 h (day 4), the cells were then cultured in maturation medium (DMEM supplemented with 10% foetal bovine serum and 5 μg/mL insulin) for 10 days.

For the MTT and Oil Red O assays the cells were incubated with YGD, *yerba maté* (at the following concentrations: 50, 75, 100, 150, 200 or 300 μg/mL), resveratrol (at the following concentrations: 10, 20 or 30 μg/mL), YGD + resveratrol (100:10 or 150:10 μg/mL), or *yerba maté* + resveratrol (150:10 or 200:10 μg/mL) during the differentiation period (from day 0 to day 4).

#### 3.2.2. Cytotoxicity Determination—MTT Assay

Cell toxicity was estimated by using the tetrazolium salt reduction test (MTT assay) on 3T3-L1 cells after exposure with the described compounds (at day 4). Briefly, the cells were cultured as described and incubated with the compounds or vehicle (0.1% DMSO) at 37 °C in 5% CO_2_. The supernatant was discarded and MTT was added (5 mg/mL in PBS). Cells were incubated for 3 h at 37 °C in 5% CO_2_, after which 100 µL 10% sodium dodecyl sulfate in 0.01 M HCl was added to each well. Samples were then incubated for 18 h at 37 °C and absorbance was measured at 540 nm in a microplate reader (Multiscan MS: Labsystems, Joensuu, Finland). Data are presented as mean ± SEM from three independent experiments (each in triplicate).

#### 3.2.3. 3T3-L1 Differentiation Assay—Oil Red O

Differentiation was evaluated using an Oil Red O staining assay. Briefly, the cells were incubated with the compounds during the differentiation period (day 0). At the end of the maturation period, the cells were washed with PBS, fixed in 4% paraformaldehyde for 30 min and incubated for 1 h with 1% Oil Red O solution [72]. After multiple water washes, the Oil Red O was dissolved in 100% isopropyl alcohol, and the optical density was measured on a microplate spectrophotometer at 540 nm. The data are presented as mean ± SEM from three independent experiments (each in triplicate).

#### 3.2.4. Quantification of the Triglyceride Content

Intracellular triglycerides content was assessed using commercial kit (LABORLAB, Guarulhos-SP, Brazil). Briefly, the cells were incubated with the compounds during the differentiation period (day 0) as previously described. At the end of the maturation period, the cells were deprived of fetal bovine serum 24 h before harvest. The absorbance at 550 nm is proportional to the concentration of triglycerides of each sample. All samples were determined in triplicate.

#### 3.2.5. RNA Extraction and cDNA Synthesis

The 3T3-L1 cells were cultured as previously described. The material used for RNA extraction was harvested at the end of the differentiation period (day 4). To stabilize and protect the RNA, all samples were stored at −80 °C in RNAlater (Qiagen, Valencia, CA, USA). Total RNA was isolated using the RNeasy tissue kit (Qiagen Valencia, CA, USA). After the extraction, ~100 ng of RNA was used for cDNA synthesis using the High Capacity cDNA Archive Kit (Applied Biosystems, Foster City, CA, USA) according to the manufacturer’s protocol.

The Mouse Adipogenesis RT^2^ Profiler™ PCR Array (SABiosciences Corporation, Frederick, MD, USA) was utilized to determine the expression profile of 84 key adipogenesis genes.

The gene array profiled the expression of 84 genes, including regulatory genes, PPARγ targets, and genes related to adipogenesis. In addition, the array included controls for human genomic DNA contamination and the reverse transcription step, a positive control for the PCR, and 5 housekeeping genes (ribosomal protein large P1 (*Rplp1*), hypoxanthine phosphoribosyltransferase 1 (*Hprt1*), ribosomal protein L13A (*Rpl13a*), lactate dehydrogenase A (*Ldha*), and beta-actin (*Actb*)).

Quantitative PCR was performed on a 7500 Real-Time PCR System (Applied Biosystems, Foster City, CA, USA), and the threshold cycle numbers were determined using RQ Study Software (Applied Biosystems, Foster, CA, USA). The reactions were performed in duplicate, and the threshold cycle numbers were averaged. The 50 µL reaction mixtures contained 25 µL Platinum SYBR Green Quantitative PCR SuperMix-UDG (Invitrogen™ Life Technologies, Alameda, CA, USA) and 100 ng of cDNA. The reactions were cycled through a preliminary UDG treatment for 2 min at 50 °C and 2 min denaturation at 95 °C, which was followed by 45 cycles of denaturation at 95 °C for 15 s, annealing for 15 s, and primer extension at 72 °C for 15 s. Subsequently, a melting point analysis of the double-stranded amplicons was performed that consisted of 40 cycles with a 1 °C decrement (15 s each) in each cycle, beginning at 95 °C. Data are presented as mean ± SD from three independent experiments (each in triplicate). The data were analyzed using web-based PCR data analysis software (SABiosciences) and normalised to the 5 housekeeping genes.

### 3.3. Statistical Analysis

The data obtained were analyzed using the SPSS 13.0 statistical Software (SPSS Incorporation Chicago, IL, USA). Comparisons between groups of data were performed using a one-way ANOVA followed by Dunnett’s Multiple Comparisons. An associated probability (*p* value) of less than 5% was considered to be significant (*p* < 0.05). For gene expression, significative down-regulation was considered when the fold regulation was ≤−2; significative up-regulation was considered when the fold regulation was ≥+2.

## 4. Conclusions

This study determined that *yerba maté*, YGD and resveratrol regulate the expression of genes that are involved in adipogenesis. Furthermore, these compounds are postulated to regulate adipogenesis through the WNT pathway, which would result in a significant repression of *Pparγ2* and *C/EBP*-α. The results also showed that resveratrol regulates a greater number of targets than *yerba maté* and YGD alone. The combination of resveratrol -yerba mate or -YGD indicates that the synergy between the compounds might favors the inhibition of adipogenesis by modulation the expression of all studied genes.
